# Unlocking high capacities of graphite anodes for potassium-ion batteries†

**DOI:** 10.1039/c9ra01931f

**Published:** 2019-07-05

**Authors:** Marco Carboni, Andrew J. Naylor, Mario Valvo, Reza Younesi

**Affiliations:** Department of Chemistry – Ångström Laboratory, Uppsala University Box 538, SE-75121 Uppsala Sweden reza.younesi@kemi.uu.se

## Abstract

Graphite is considered a promising candidate as the anode for potassium-ion batteries (KIBs). Here, we demonstrate a significant improvement in performance through the ball-milling of graphite. Electrochemical techniques show reversible K-intercalation into graphitic layers, with 65% capacity retention after 100 cycles from initial capacities and extended cycling beyond 200 cycles. Such an affinity of the graphite towards storage of K-ions is explained by means of SEM and Raman analyses. Graphite ball-milling results in a gentle mechanical exfoliation of the graphene layers and simultaneous defect formation, leading to enhanced electrochemical performance.

## Abstract 2

Ball-milling of graphite results in graphene layer exfoliation and defect formation, leading to enhanced performance as a K-ion battery anode.

## Graphical abstract

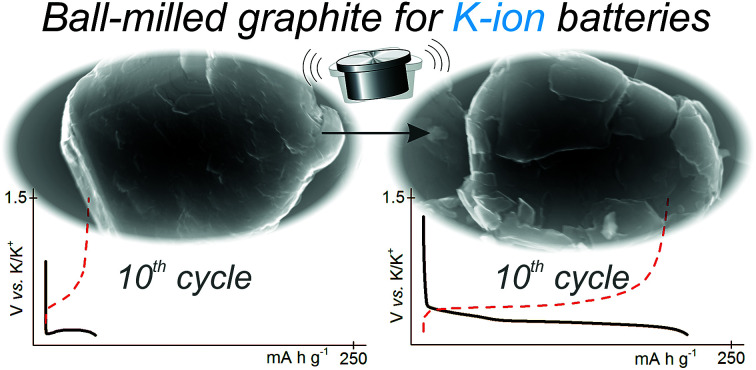

## 1. Introduction

The growing interest in potassium-ion batteries (KIBs) arises from the number of interesting aspects of KIBs compared to lithium-ion batteries (LIBs). The abundance of potassium in the earth's crust (*i.e.* around 900 times higher than lithium), its availability and low cost, are significant factors that can lead towards the commercialisation of KIBs in the future.^1,2^ Moreover, expensive copper current collectors, as used in LIBs, can be replaced by aluminium, since potassium does not alloy with it at low potentials.^3^ In addition, potassium exhibits a lower positive charge density, offering increased ion mobility in electrodes and electrolytes, ideal for fast charging applications.^2^ Materials and methodologies already extensively used in LIB production can be applied to KIBs. Analogous K-based electrolytes (*e.g.* KPF_6_, KFSI), anodes (*e.g.* C, TiO_2_, P) and cathodes (*e.g.* Prussian blue analogues, layered oxides, polyanionic compounds and organic materials) have already been reported for storage by intercalation, alloying and conversion mechanisms.^4–8^ However, potassium does suffer from higher atomic weight and ionic radius compared with Li, presenting challenges in terms of gravimetric and volumetric energy densities. This is despite potassium having a very similar redox potential to that of Li (−2.93 V *vs.* SHE).^4^

The highly reversible electrochemical K-insertion into graphite showed by Komaba *et al.*^4^ and Jian *et al.*^5^ has brought about renewed enthusiasm towards graphitic materials as anodes in KIBs.^9,10^ Great efforts have been made to understand the formation mechanism of the so called K-Graphite Intercalation Compounds (K-GICs) and a combination of theoretical and experimental studies has revealed that the reversible sequence C–KC_36_–KC_24_–KC_8_ is the stage pathway followed by K-GICs during discharge/charge processes.^5,11^ Experimental values of 273 and 244 mA h g^−1^ after the first discharge (potassium insertion)^4,5^ are in good agreement for the theoretical specific capacity for KC_8_ of 279 mA h g^−1^. Unfortunately, many graphite-based electrodes suffer from low capacity retention on extended cycling. A large volume expansion during the insertion of potassium has been claimed to be the main cause of the capacity drop,^4,5^ although the choices of cell configuration, binders, separators and electrolytes are also critical to prolong the lifetime of these cells.^4,12^

Here we report the substantial gains in electrochemical performance achieved by using ball-milled graphite as an anode for KIBs. The physical properties of the graphite are evaluated using scanning electron microscopy (SEM) and Raman spectroscopy, while electrochemical techniques are used to determine cycling performance and to shed light on potassium insertion mechanisms and solid electrolyte interphase (SEI) stability.

## 2. Experimental methods

### 2.1 Materials and electrode preparation

Electrodes were prepared from commercially available graphite by two different methods for testing as anodes for potassium-ion batteries. In the first one, 0.200 g of commercial graphite powder (SLP-30, Timcal SA) and a 10% solution of polyvinylidene fluoride (PVDF, Arkema Kynar® FLEX 2801) in *N*-methyl-2-pyrrolidone (NMP, Sigma Aldrich) were mixed with a graphite : PVDF ratio of 9 : 1 w/w using an agate mortar and pestle for 30 minutes. The resulting slurry was cast onto a copper foil current collector. The electrode was dried in a ventilated oven at 100 °C for 1 h and subsequently punched into 10 mm-diameter discs. Before cell assembly, these pristine graphite (PG) electrodes were further dried at 120 °C under vacuum for 12 h. For the second preparation method, the same materials, weight and weight ratios were adopted and placed in a 60 ml zirconia jar together with two balls (*ø* = 20 mm, average weight = 25.55 g each) made by the same material. The graphite and PVDF solution was thoroughly mixed by ball-milling (*i.e.* ball-milled graphite – BMG) for 1 h at 150 rpm in air, using a RETSCH PM 4 planetary ball mill. The resulting slurry was cast on a copper foil and cut and dried as described above. The active material mass loading was 1.40 ± 0.13 mg cm^−2^ for both types of electrode.

Counter and reference electrodes were prepared by pressing a piece of metallic potassium (Sigma Aldrich) onto a copper foil in an argon-filled glove box (H_2_O and O_2_ < 1 ppm). The Cu foil with the pressed potassium was then cut into discs with a diameter of 13 mm.

The electrolyte was prepared by dissolving potassium hexafluorophosphate (KPF_6_, Sigma Aldrich) in a mixture of 1 : 1 v/v ethylene carbonate and diethyl carbonate (EC and DEC respectively, Sigma Aldrich) to obtain a 0.8 M concentration. Prior to electrolyte preparation, KPF_6_ was dried overnight under vacuum at 120 °C, while the EC : DEC mixture was used after drying/storage on regenerated 3 Å molecular sieves (Sigma Aldrich) for at least 15 days in a argon-filled glovebox (H_2_O and O_2_ < 1 ppm).

### 2.2 Cell assembly

Half-cells were assembled in rectangular polymer-coated aluminium pouches (10 × 12 cm) using either the pristine graphite (PG) or ball-milled graphite (BMG) as working electrode and metallic potassium on copper (Cu/K) as combined reference and counter-electrode. A Solupor® 3P07A separator with the thickness of 20 μm and porosity of 83% was cut into *ø* = 15 mm disc, placed between the electrodes in a pouch and then wetted with 0.1 mL of electrolyte using a micro fixed volume pipette. The pouches were finally sealed reducing the pressure down to 20 mbar. Symmetrical cells and three-electrode cells were also assembled in the same way, by replacing graphite with a Cu/K electrode and using another Cu/K electrode as reference, respectively.

### 2.3 Electrochemical measurements

Cyclic voltammetry (CV) was carried out with a half-cell configuration using a VMP3 (Bio-Logic) potentiostat, by applying a scan rate of 0.1 mV s^−1^ and a potential cut-off of 0.01 and 1.80 V *vs.* K^+^/K. Galvanostatic cycling was performed with the same equipment for the three-electrode cell tests, at two different specific current densities, 25 and 250 mA g^−1^, with a voltage cut-off of 0.01 and 1.50 V *vs.* K^+^/K. Half-cells and symmetrical cells were cycled using an Arbin battery cycler with rates of 25 mA g^−1^ and 0.1 mA cm^−2^, respectively. The voltage limitations for the half-cells were 0.01 and 1.50 V *vs.* K^+^/K, while the capacity limitation for the symmetric cells was 0.5 mA h cm^−2^. Pause tests^13,14^ were also performed on the half-cells, alternating galvanostatic cycles (at 25 mA g^−1^) with three extended pauses (OCV periods) for 4, 8 and 4 days, respectively.

### 2.4 Electrode characterisation

Raman spectroscopy was performed on a Renishaw inVia Raman microscope using an excitation wavelength of 532 nm from a laser diode with a maximum power of 500 mW. The laser beam was focused on the surface of the as-prepared graphite electrode specimens *via* a 50× magnification objective. A constant laser power nominally corresponding to 0.5% of its maximum value was utilized for the analyses. A preliminary calibration of the spectrometer was performed by means of a Si wafer to obtain a characteristic reference peak at 520.6 cm^−1^. 40 cumulative acquisitions with a measuring time of 20 s were run for each spectrum between 200 cm^−1^ and 3200 cm^−1^. The exposure to the laser beam was minimized in between subsequent measurements to avoid any possible degradation of the sample surface.

The surface morphologies of the as-prepared electrodes were investigated by means of a Field Emission Scanning Electron Microscope (FE-SEM, Zeiss LEO1550) *via* a dedicated In-Lens secondary electron detector and employing magnifications of 1000× and 20 000× with an operation voltage of 10 keV. The cast electrode samples were mounted on aluminium stubs using double-sided adhesive conductive copper tape.

## 3. Results and discussion

**Fig. 1:**
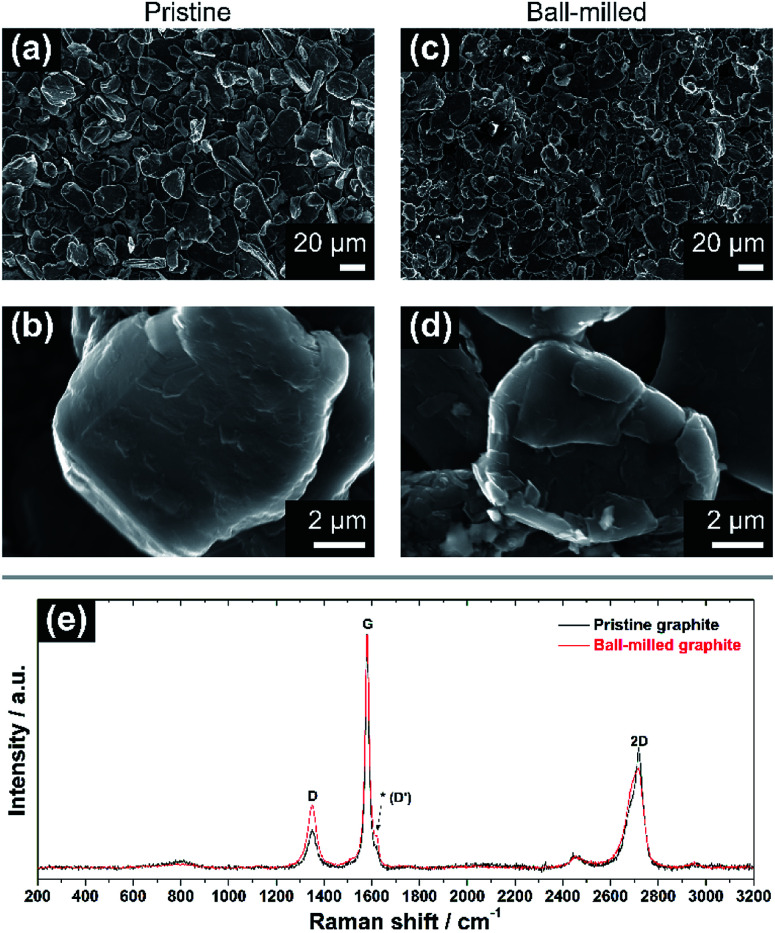
(a and b) SEM images of the pristine graphite (PG) electrode at low (1000×) and high (20 000×) magnification. (c and d) SEM micrographs of ball-milled graphite (BMG) electrode at low and high magnification. (e) Normalized Raman spectra of pristine (black) and ball-milled graphite (red). Note the increase of the relative intensity of the ‘D’ band in (e) after ball-milling, as well as a more prominent signal at ∼1620 cm^−1^ (see asterisk).

The morphological and vibrational features of electrodes of pristine graphite (PG) and ball-milled graphite (BMG) are presented in Fig. 1. SEM images of PG particles in Fig. 1a and b display large and uneven particles with a wide particle size distribution ranging from a few micrometres to 30–40 μm. At high magnification (Fig. 1b), it is possible to observe the compact structure of the graphite particles, and even signs of the layered arrangement. The mild ball-milling process mainly induces two different effects as determined from Fig. 1c and d. The size of the graphite particles is reduced to about 20–25 μm, and it is clear that the ball-milling results in the formation of a more defective particle surface, which appears very uneven compared with the pristine particles.

Raman spectroscopy spectra of both PG and BMG electrodes are show in Fig. 1e. The spectrum of the PG electrode presents typical features of defective graphitic carbons, namely two main peaks at 1355 and 1580 cm^−1^, or so-called D and G bands, respectively.^13,14^ The G band is related to first order Raman signal of sp^2^-hybridized carbon species and this vibration mode has E_2g_ symmetry involving in-plane bond-stretching movement of all pairs of C atoms with sp^2^ hybridization in both rings and chains.^14,15^ The interpretation of the D band is less straightforward and, although it is ascribed to the A_1g_ breathing mode of sp^2^ C atoms, understanding of its features results complex according to solid-state theory.^14^ This A_1g_ mode is forbidden in perfect graphite and activates only in presence of disorder.^15,16^ The intensity ratio of the G and D bands (*i.e. I*_G_/*I*_D_) ties well with the degree of disorder here due to edge defects in the graphitic framework.^16^ The ‘2D’ band at ∼2700 cm^−1^ refers to the second order excitation of the D peak and consists of a main component at ∼2720 cm^−1^ and a shoulder at lower wavenumbers, with roughly 1/2 and 1/4 of the intensity of the G peak, respectively.^14,16,17^ The simultaneous presence of these two components confirms the existence of multi-layered graphitic frameworks. The spectrum of the BMG electrode displays the same peaks of PG, however, their relative intensities are visibly changed. In particular, the decreasing *I*_G_/*I*_D_ ratio highlights an increment of defects in the graphite particles, induced by the size reduction of the graphitic domains due to the ball-milling (see ESI†). Another feature becomes also more pronounced after ball-milling around ∼1620 cm^−1^. The latter is often referred to as D′ peak and is characteristic of defected graphite.^14^ Furthermore, a broader shoulder of the band at ∼2700 cm^−1^ confirms a soft exfoliation of the graphite, without completely isolating the graphene layers from the main carbon domains, which is in agreement with the SEM analysis.

**Fig. 2:**
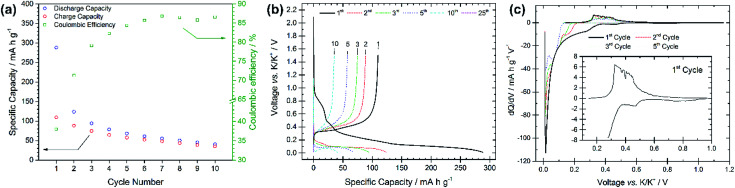
(a) Specific capacity *vs.* cycle number for the pristine graphite electrode galvanostatically cycled at 25 mA g^−1^ with potential limitations of 0.01 V and 1.5 V *vs.* K/K^+^. (b) Voltage profiles *vs.* specific capacity for selected cycle numbers from the same test. (c) d*Q*/d*V* plots for cycles 1, 2, 3 and 5 for the same test.

The electrochemical performance of graphite electrodes is evaluated here using half-cell configurations with potassium metal as the counter electrode. A symmetrical cell using potassium metal as both electrodes was used to demonstrate that the plating-stripping of potassium experiences an overpotential of approximately ±0.1 V for the first 10–15 cycles (150 h), after which the polarization gradually increases to about ±0.2 V (Fig. S1†). This demonstrates that potassium metal electrodes provide a reasonably low and acceptable polarization, thus it can be used as the counter electrode in half-cells. This is an interesting finding compared to lithium and sodium metals which have shown to be unsuitable counter electrodes because of their low efficiencies and high overpotentials, influencing the electrochemical performance of working electrodes.^18–21^ Furthermore, the BMG electrode tested in three-electrode cells with potassium metal as both counter and reference electrodes shows suitable stability of the counter electrode especially at low rates (Fig. S2†). The electrochemical performance of the as-received graphite, cast as an electrode, is presented in Fig. 2. The PG electrode offers a specific capacity of 285 mA h g^−1^ during the first discharge (potassium insertion) and a poor reversible capacity of 115 mA h g^−1^ upon charging (potassium extraction). Subsequent cycling leads to extensive capacity fade, with cell failure after only 25 cycles. Nevertheless, the coulombic efficiency (CE, [*C*_ch_/*C*_dis_] × 100%), only 41% for the first cycle, increases upon cycling, while remaining still far from previously reported efficiencies of ≥95%.^10^ Four voltage plateaux are visible (Fig. 2b) on the first discharge at approximately 0.75, 0.45, 0.25 and 0.1 V, which are more easily observed in the d*Q*/d*V* plot in Fig. 2c as definitive peaks. The peaks at higher voltages are visible only on the first cycle and are ascribed to SEI formation, while the lower voltage features are assigned to potassium insertion into graphite.

**Fig. 3:**
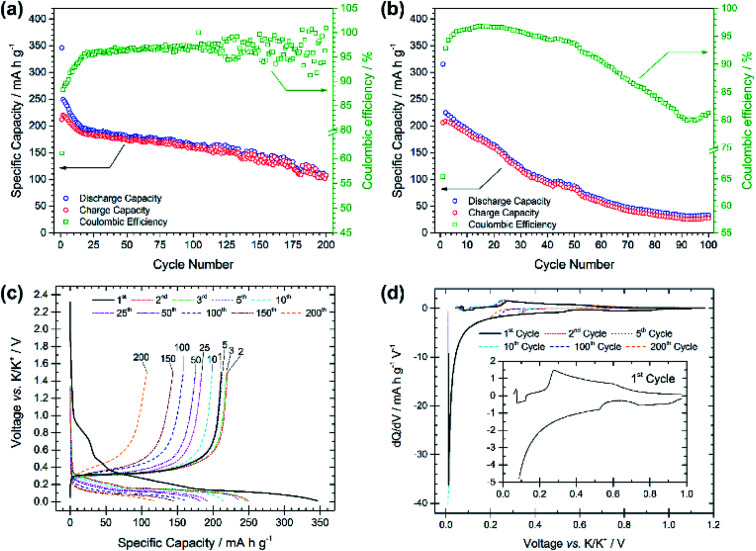
Specific capacity *vs.* cycle number for the ball-milled graphite (BMG) electrode galvanostatically cycled at (a) 25 mA g^−1^ and (b) 250 mA g^−1^ with potential limitations of 0.01 V and 1.5 V *vs.* K/K^+^. (c) Voltage profiles *vs.* specific capacity for selected cycle numbers from the test at 25 mA g^−1^. (d) d*Q*/d*V* plots for cycles 1, 2, 5, 10, 100, and 200 from the test at 25 mA g^−1^.

Compared with the PG electrode, the BMG electrode shows significantly improved electrochemical performance at the same applied current density (see Fig. 3a). The BMG exhibits an initial discharge capacity of 345 mA h g^−1^, and charge capacity of 211 mA h g^−1^, corresponding to a CE of 61% for the first cycle. This demonstrates a greater extent of potassium insertion into graphite with higher reversibility than for PG. A jump in charge capacity to 220 mA h g^−1^ is observed for the second cycle, and, upon further cycling, discharge capacities higher than 150 and 100 mA h g^−1^ are achieved after 100 and 200 cycles, respectively. Moreover, a CE of 88% is calculated for the second cycle, which then soon yields values of ≥95%. Fig. 3b shows the electrochemical performance of the BMG at a higher current density of 250 mA g^−1^, corresponding to a C-rate of ∼C/1.1. Accordingly, the BMG electrode cycled at this C-rate shows initial promising behaviour with a charge capacity of 209 mA h g^−1^ in the second cycle and CE values higher than 90% for 50 cycles. Nevertheless, the capacity fade on extended cycling is evident, yielding only a 36% capacity retention after 50 cycles and 12% after 100 cycles. The voltage profile for the first discharge in Fig. 3c appears very similar to that for the PG electrode, with a number of plateaux corresponding to SEI formation and potassium insertion. This is evident also from d*Q*/d*V* plots (Fig. 3d) which confirm similar processes occurring for both the BMG and PG. Cyclic voltammetry measurements (Fig. S3†) complement d*Q*/d*V* plots showing peaks at between 1.10 and 0.60 V corresponding to SEI formation, together with a prominent feature at 0.40–0.01 V corresponding to potassium insertion into graphite. SEI formation is confirmed to mainly occur only during the first cycle, since in subsequent cycles, only the intercalation peak is detected during reduction.

These results demonstrate a rather high electrochemical reversibility of the BMG and clearly demonstrate the role of the ball-milling process, which enables close to the theoretical capacities and only a moderate capacity fade upon prolonged cycling. Extensive SEI formation appears to occur mainly during the first discharge, after which the insertion/extraction mechanism is seen to be reasonably reversible.

**Fig. 4:**
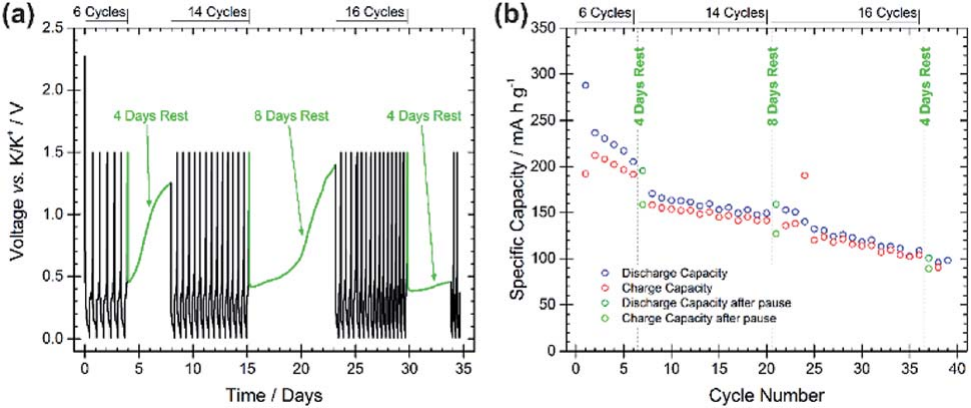
(a) Voltage profile of a pause test at 25 mA g^−1^ using BMG as the working electrode. Potential limitations of 0.01 V and 1.5 V *vs.* K/K^+^ were used with the following cycling regime: 6 cycles, rest 4 days, 14 cycles, rest 8 days, 16 cycles, rest 4 days, continued cycling. (b) Specific capacity for galvanostatic discharge/charge cycles during the pause test.

The stability and evolution of the SEI was studied by means of galvanostatic pause tests, as shown in Fig. 4, subjecting the BMG electrode to alternating periods of galvanostatic cycling and open-circuit voltage (OCV) measurements.^22^ Comparing the discharge capacities of the cycles before and after the pauses allows for the investigation of SEI behaviour during the OCV period. A decrease in the discharge capacity implies a continued spontaneous passivation of the electrode surface during the OCV period, whereas an increase indicates the dissolution of the SEI into the electrolyte and consequent reformation of the SEI. Discharge capacities of 205 and 195 mA h g^−1^ (−4.9%) are measured at the 6^th^ and 7^th^ cycles, before and after the first pause of four days, indicating that a spontaneous passivation effectively occurred. This is in agreement with the typically large capacity decay observed in the first cycles for the BMG electrode. The rapid capacity fade can be attributed to continued passivation of the electrode surface. Discharge capacities of 149 and 159 mA h g^−1^ (+6.7%) were recorded before and after the second pause, and 108 and 101 mA h g^−1^ (−6.5%) before and after the third pause. The increase in capacity after the second pause suggests that some dissolution of the SEI has occurred during the rest, which is being reformed on the subsequent discharge. However, the decrease after the third pause indicates still continued SEI formation even in later cycles.

## 4. Conclusions

Overall, we have demonstrated the key role that ball-mill processing has upon the electrochemical performance of graphite anodes in potassium-ion half-cells. High specific capacities of more than 200 mA h g^−1^ are reported after 10 cycles, at which point the as-received graphite had already lost 50% of its initial capacity. Subsequent extended cycling beyond 200 cycles is achieved with coulombic efficiencies reaching on average 98%. This substantially improved performance of the ball-milled graphite is explained through SEM and Raman analyses, which confirm a gentle mechanical exfoliation of the graphene layers with concomitant formation of structural defects. The high stability of the potassium metal counter electrode has been verified in half-cells, yet with some instability or continued growth of the SEI on these graphite electrodes, as indicated by electrochemical pause tests. Graphite ball-milling should therefore be employed as a standard method for fabricating these potassium-ion anodes, in order to provide enhanced performances.

## Conflicts of interest

There are no conflicts to declare.

## Supplementary Material

RA-009-C9RA01931F-s001
