# Supplementary material for: Unlocking high capacities of graphite anodes for potassium-ion batteries
Source: RSC Adv. 2019 Jul 5;9(36):21070–4. doi: 10.1039/c9ra01931f (PMC9065985; doi:10.1039/c9ra01931f)
Supplement: RA-009-C9RA01931F-s001 [file RA-009-C9RA01931F-s001.pdf]

Electronic Supplementary Material (ESI) for RSC Advances.
This journal is © The Royal Society of Chemistry 2019

# Unlocking high capacities of graphite anodes for potassium-ion batteries

*Marco Carboni,[a] Andrew J. Naylor,[a] Mario Valvo [a] and Reza Younesi [a]*

*[a]Department of Chemistry - Ångström Laboratory, Uppsala University, Box 538, SE-75121, Uppsala, Sweden*

# Supporting Information

## Calculation of $I_D/I_G$ ratio

**Table S1.** Characteristic features extracted from the Raman analysis of pristine graphite (PG) and ball-milled graphite (BMG).

| Graphite sample | *I(D)/I(G)* | $L_a$ (*nm*) |
|---|---|---|
| PG | *0.1651* | *30.04* |
| BMG | *0.2699* | *18.38* |

The ratio of the intensities of the *D* and *G* bands in graphite can be correlated to a characteristic graphitic cluster diameter also referred to as in-plane correlation length, $L_a$, via the following equation:[1,2]

$$\frac{I(D)}{I(G)} = \frac{C(\lambda)}{L_a} \qquad \text{(S1)}$$

where *C*(l) is a variable scaling coefficient depending on the laser excitation wavelength, l, which can be calculated via the following formula:

$$C(\mathrm{l}) \approx C_0 + \mathrm{l}C_1 \qquad \text{(S2)}$$

where $C_0$ and $C_1$ were estimated in a previous work[3] to be -126 Å and 0.033, respectively. Hence, the utilization of a laser source with l = 5320 Å yields *C*(5320) = 49.56 Å ≈ 49.6 Å for our Raman analyses of pristine graphite (PG) and ball-milled graphite (BMG). Accordingly, the corresponding values of $L_a$, calculated via eq. (S1) and reported in Table S1, indicate a clear decrease of the typical graphitic cluster size as a result of the ball-milling process for BMG, which concurrently leads to an enhanced formation of structural defects.

**Plating-stripping of potassium metal**

The plating-stripping performance of potassium metal assessed to determine whether or not any overpotential is exhibited for the potassium counter-electrode in half-cells. Figure S1 shows the voltage profile of a symmetrical cell using potassium metal as both electrodes.

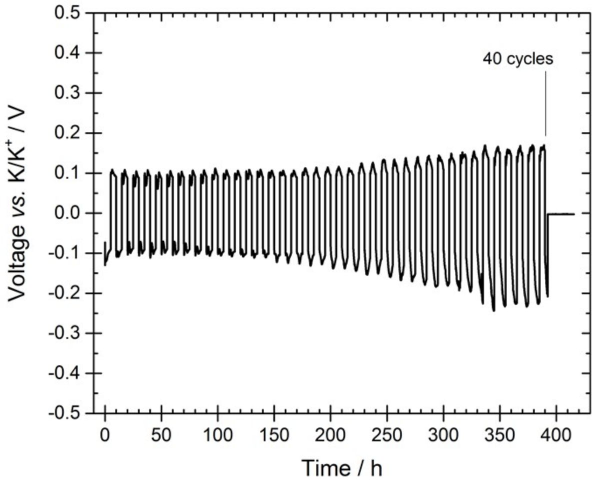


Figure S1. Voltage profile for potassium plating and stripping reactions in a symmetrical cell configuration using two potassium metal electrodes. An electrolyte of 0.8 M $KPF_6$ in EC/DEC (1:1 v/v) was used and the cell tested with a current density of 0.1 mA $cm^{-2}$ and a capacity cut-off of 0.5 mA h $cm^{-2}$.

**Three-electrode cell testing with BMG electrode**

The electrochemical performance of BMG was tested in a three-electrode cell configuration using an additional potassium metal electrode as reference to investigate what extent of the capacity fading is as a result of passivation at the counter electrode. Voltage profiles for the potassium counter electrode are shown in Figure S2a over 60 cycles at current densities of 25 mA $g^{-1}$ and 250 mA $g^{-1}$. The potassium counter electrode appears stable over 60 cycles at 25 mA $g^{-1}$, suffering only from a slight polarization of ±0.01 V, which decreases with continued cycling. The contributions of the counter-electrode are significantly larger at the higher rate, and increase during cycling from approximately ±0.07 to ±0.1 V. This result indicates that the resistance of the K-metal surface can be neglected at low rates, while its contribution to possible cell failure has to be taken into account when higher currents are applied. The corresponding load curved are shown in Figure S2b.

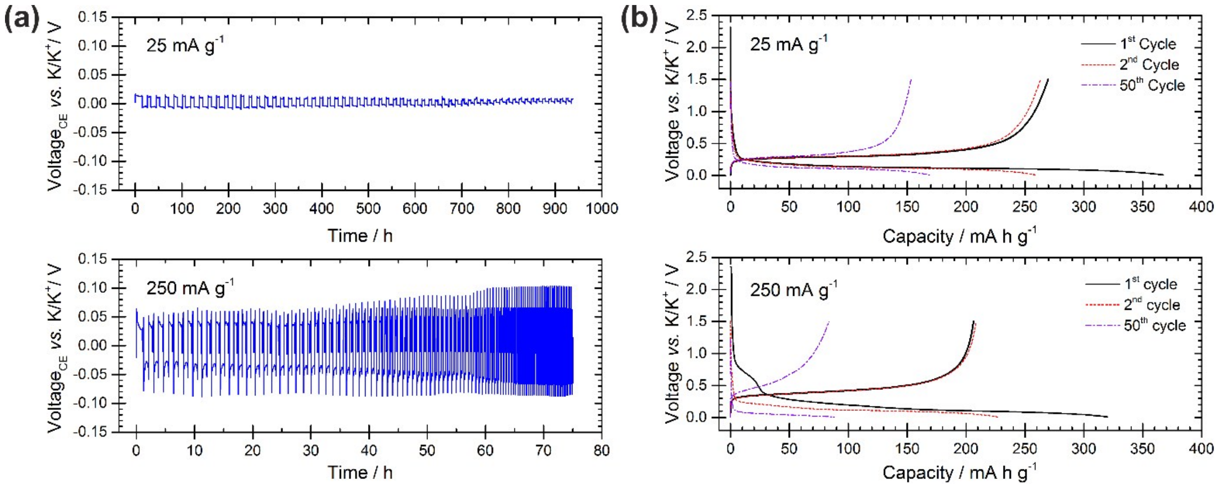


Figure S2. (a) Voltage profiles over 60 cycles of the counter electrode (potassium metal) in a three-electrode cell employing BMG as the working electrode and potassium metal as a reference electrode at current densities of 25 mA $g^{-1}$ (top) and 250 mA $g^{-1}$ (bottom), corresponding to C-rates of approximately ~C/1.1 and ~C/11, respectively, based on a theoretical capacity of 279 mA h $g^{-1}$. Voltage limitations of 0.01 V and 1.5 V *vs.* $K/K^+$ were used. (b) Voltage profiles (working electrode) *vs.* specific capacity for $1^{st}$, $2^{nd}$ and $50^{th}$ cycles for the three-electrode cells at 25 mA $g^{-1}$ (top) and 250 mA $g^{-1}$ (bottom).

### Cyclic voltammetry of BMG electrode

Cyclic voltammetry measurements (Figure S3) of BMG as the working electrode in a half-cell were performed. During the first cathodic sweep, a broad peak is visible between 1.10 and 0.60 V together with a prominent feature at 0.40-0.01 V comprising a main peak at low voltage and a shoulder at slightly high voltage (0.40-0.30 V). The broad peak and the shoulder can be attributed to SEI formation, while the peak at lower potential is assigned to the potassium insertion into graphite, although no stage processes of C–$KC_{36}$–$KC_{24}$–$KC_8$ formation can be identified. On the reverse scan, three peaks appear in a broad anodic band at 0.40, 0.50 and 0.75 V and can be ascribed to sequential transitions among the K-GICs.[4–6] In the following cycles, only the intercalation peak is detected during reduction, while the broad peak and shoulder disappear. This confirms that the SEI formation primarily occurs during the first reduction and passivates the electrodes thereafter. The subsequent anodic sweeps show a reversible and consistent behaviour of the K-GICs over many cycles.

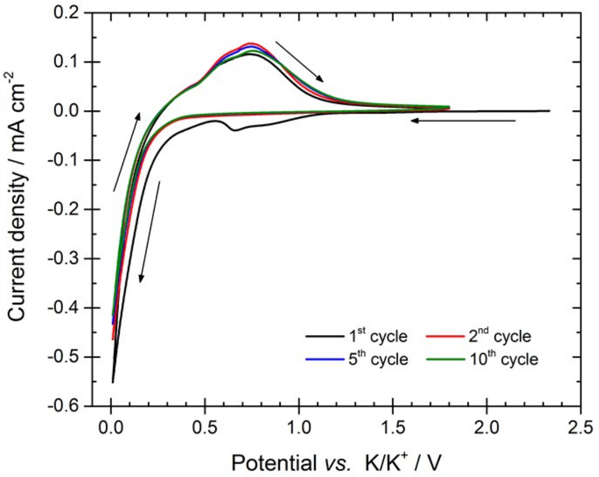


Figure S3. Cycling voltammetry of the BMG electrode with potassium metal counter electrode and electrolyte of 0.8 M $KPF_6$ in EC/DEC. Voltage limits of 0.01 and 1.80 V vs. $K/K^+$ and a scan rate of 0.1 mV $s^{-1}$ were used.


## References

1 A. C. Ferrari and J. Robertson, *Phys. Rev. B*, 2000, **61**, 14095–14107.

2 F. Tuinstra and J. L. Koenig, *J. Chem. Phys.*, 1970, **53**, 1126–1130.

3 M. Endo and M. A. Pimenta, *Phys. Rev. B - Condens. Matter Mater. Phys.*, 1999, **59**, R6585–R6588.

4 Y. Li, R. A. Adams, A. Arora, V. G. Pol, A. M. Levine, R. J. Lee, K. Akato, A. K. Naskar and M. P. Paranthaman, *J. Electrochem. Soc.*, 2017, **164**, A1234–A1238.

5 J. Zhao, X. Zou, Y. Zhu, Y. Xu and C. Wang, *Adv. Funct. Mater.*, 2016, **26**, 8103–8110.

6 Z. Xing, Y. Qi, Z. Jian and X. Ji, *ACS Appl. Mater. Interfaces*, 2017, **9**, 4343–4351.
